# CDC Grand Rounds: Evidence-Based Injury Prevention

**Published:** 2014-01-03

Approximately 5.8 million persons die from injuries each year, accounting for 10% of all deaths worldwide (1). In the United States, 180,000 persons die each year from injuries, making the category the country’s leading cause of death for those aged 1–44 years and the leading cause of years of potential life lost before age 65 years (2). Injuries also result in 2.8 million hospitalizations and 29 million emergency department visits each year in the United States. Motor vehicle crashes, falls, homicides, suicides, domestic violence, child maltreatment, and other forms of intentional and unintentional injury affect all strata of society, with widespread physical, mental, and reproductive health consequences. Injuries and violence affect not only individuals, but also families and communities, producing substantial economic and societal burdens related to health-care costs, work loss, and disruption of education. The estimated annual U.S. cost in medical expenses and lost productivity resulting from injuries is $355 billion (2).

As is true in most areas of public health, to effectively prevent injuries, injury and violence prevention strategies and interventions should be tested in real-world settings. Real-world settings also can be fertile laboratories for generating new interventions and prevention strategies. Community input to help identify and prioritize problems for which interventions should be developed, propose interventional models, and test, refine, and adapt interventions can help ensure relevance, feasibility, acceptability, scalability, and sustainability.

Translating injury and violence prevention evidence into action in the United States depends on coordination among federal, state, and local agencies, and partnerships in the research and practice communities. In 2010, CDC published a compendium of 22 effective interventions from around the world aimed at prevention of falls among older adults (3), then funded the translation of some of these strategies into programs for specific communities and delivery systems. Three programs to prevent falls that were highlighted in the compendium currently are being piloted in Colorado, New York, and Oregon (3). The following two case studies on suicide and alcohol-impaired driving are examples of using an evidence-based approach to injury and violence prevention to improve public health policy and practice.

## Case Study 1: Using Science to Guide Suicide Prevention Activities in Oregon

During the past 10 years, the U.S. suicide rate has increased approximately 10%, despite greater recognition of the problem and expansion of antidepressant use (4). For every suicide death, approximately 11 suicide attempts are made, and many other persons have suicidal thoughts. One theorist has suggested that there are three key factors leading to suicide: 1) “thwarted belongingness,” or feelings of alienation despite trying to connect with others; 2) “perceived burdensomeness,” or feeling like a burden to others; and 3) “the acquired ability to enact lethal self-injury,” or desensitization to pain and death from repeated exposure. The last factor is supported by the observation that the risk factor most strongly associated with dying by suicide is having attempted suicide previously; a pattern of increasing lethality of attempts is observed among some suicide decedents (5).

In 2010 in Oregon, a total of 685 deaths were attributed to suicide, more than in 2009 and more than the number of deaths attributed to motor vehicle crashes. Suicide was the state’s eighth leading cause of death, and the rate of death by suicide among men was almost four times the rate among women. The highest suicide death rates were observed in men aged ≥75 years. To address the high suicide rate, the Public Health Division of the Oregon Health Authority, along with other state agencies and representatives from 13 communities throughout Oregon, created a suicide prevention plan for older adults. Development of the plan was funded by CDC and the Substance Abuse and Mental Health Services Administration.

Surveillance data from the National Violent Death Reporting System (NVDRS) was important to development of the plan. NVDRS is a registry of deaths by suicide, homicide, legal intervention, and undetermined intent that links data from multiple sources, including death certificates, medical examiners, law enforcement, and crime laboratories. Through NVDRS, public health practitioners and researchers have access to data regarding the circumstances surrounding reported deaths that are not available from the National Vital Statistics System. NVDRS has been in operation since 2002 and is currently implemented in 18 states, including Oregon.

In 2009, NVDRS data for the 640 reported suicide deaths in Oregon indicated that 209 (33%) of the decedents had experienced a depressed mood, and 268 (42%) had disclosed suicidal intent (Table). Persons aged 20–44 years were most likely to have disclosed suicidal intent (50%), followed by those aged ≥65 years (40%), aged 45–64 years (38%), and aged 10–24 years (37%).

Whereas substantial percentages of suicide decedents in younger age groups had experienced alcohol or substance abuse (e.g., 34% of those aged 20–44 years) and relationship problems (e.g., 48% of those aged 20–44 years and 36% of those aged 10–24 years), chronic disease or declining health was more prevalent (68%) among suicide decedents aged ≥65 years (Table). Additional findings exclusively regarding decedents aged ≥65 years (prevalences in other age groups were not assessed) indicated that 44% had lived alone, and only 17% had visited a health-care provider in the 30 days before death, suggesting a need for community intervention to reduce social isolation and use of health-care encounters as intervention venues. The substantial prevalence of disclosed suicidal intent also supported the idea that sensitizing health-care and social-service providers to the possibility of disclosure and giving them guidance regarding how to respond might be worthwhile interventions.

The Oregon Older Adult Suicide Prevention Plan (6) has helped raise awareness about the risk for suicide among older persons. Suicide prevention interventions have been integrated into other services provided to older adults and also have been included in broader agency discussions about promoting healthy aging. As one result, Oregon’s state health department has collaborated with Oregon Health and Sciences University to develop a web-based training program for primary-care providers on recognition and management of suicide risks among older adults.

## Case Study 2: From Evidence to Policy in Alcohol-Impaired Driving

In 2011, alcohol-impaired driving resulted in almost 10,000 traffic deaths in the United States, accounting for one third of all traffic-related deaths (7), approximately 27 deaths per day. An analysis of data from 2010 found that alcohol-related traffic deaths cost $65 billion for that year alone (8). A conservative estimate is that one in 10 persons in the United States will be involved in an alcohol-related crash in their lifetime. Blood alcohol content (BAC), the measure of alcohol in a person’s bloodstream as detected by blood, breath, or urine testing, has been found to have a direct and dose-response effect on driving performance (9).

In 1939, Indiana became the first state to implement a presumptive BAC limit for impaired driving of 0.15% for drivers. By the 1950s, many other states followed, setting their BAC limit at 0.15% at the recommendation of the American Medical Association. By the 1960s, states began lowering their BAC limit from 0.15% to 0.10%, as scientific evidence mounted regarding the relationship between driver BAC and fatal crashes. In 1980, Utah became the first state to lower its limit to 0.08%. By 1992, the National Highway Traffic Safety Administration had proposed that all states adopt 0.08% BAC laws, and in 1998, a legislative proposal was introduced in Congress that would have required states to enact and enforce 0.08% BAC laws or face cuts in highway funding (10). That proposal failed and, instead, grants were offered to states that lowered their BAC limits to 0.08%; however, only three states did so.

In the 1990s, only four published studies had demonstrated the effectiveness of 0.08% BAC laws in reducing traffic fatalities. In 1999, a Government Accountability Office report concluded that the evidence did not conclusively establish that 0.08% BAC laws, by themselves, resulted in reductions in the number and severity of traffic crashes (11).

Subsequently, CDC and the Community Preventive Services Task Force began a systematic review of the effectiveness of 0.08% BAC laws (12). The results of nine studies that met the quality criteria set by the task force demonstrated a median 7% decline in fatalities in states with 0.08% BAC laws. It was estimated that if all states had 0.08% BAC laws, 400–600 lives could be saved annually. The task force concluded that 0.08% BAC laws were effective in reducing alcohol-related traffic fatalities and recommended enactment of these laws based on strong evidence (13). Shortly afterward, a bill was approved and subsequently signed into law on October 23, 2000, that included cuts in highway funds for states without 0.08% BAC laws, based in part on the available scientific evidence demonstrating lives could be saved. By 2004, all U.S. states had enacted 0.08% BAC legislation (14). However, the impact on reducing fatalities was not realized until several years later. Self-reported episodes of drinking and driving declined from 161 million in 2006 to 112 million in 2010, and death rates from alcohol-impaired driving have shown similar declines, with steep reductions since 2005 (7).

Additional work is needed to further reduce the incidence of fatalities related to alcohol-impaired driving in the United States, including supporting and promoting other interventions such as use of ignition interlocks and sobriety checkpoints, enforcement of primary seatbelt laws and reduction of binge drinking (15), and assessing the evidence of the impact on traffic fatalities in the United States by lowering the BAC limits even further, to 0.05%, which is already the legal limit in nearly half of all countries (16,17).

## The Future of Injury and Violence Prevention

Most events resulting in injury, death, or disability are predictable, and therefore preventable. An important contemporary challenge in injury prevention is the need to make the best use of technologies that can prevent injuries at the personal and population level, while mitigating hazards resulting from technological advances (e.g., distracted driving).

Expanded use of the Internet and social media can provide platforms to disseminate evidence-based injury prevention information. Evaluation research and community-based studies are needed to assess the effects of such communications on progress toward the ultimate goals of preventing injuries and deaths.

Community prevention efforts can attain maximum impact by recognizing that injury and violence prevention are core components of public health. Injury prevention practice can inform research, much like research informs clinical practice, and the growth and education of the next generation of practitioners and researchers needs to be ensured through training (18). Injury prevention efforts should be visible, with their value documented to ensure accountability and increase impact in communities. Innovative solutions to injury problems should be pursued, and opportunities to link clinical medicine and public health should be fostered (19). As the U.S. population becomes older and more ethnically diverse, the additional challenges of language, access to health-care information, and limited public health resources for injury and violence prevention will grow more pronounced.

### Reported by

*Linda C. Degutis, DrPH, Office of the Director; David A. Sleet, PhD, Div of Unintentional Injury, National Center for Injury Prevention and Control, CDC. Melvin Kohn, MD, Oregon Public Health Div. Georges Benjamin, MD, American Public Health Assoc. Nicole Cohen, MD, John Iskander, MD, Office of the Director, CDC.* ***Corresponding contributor:*** *David A. Sleet, dsleet@cdc.gov, 779-488-4699.*

## Figures and Tables

**TABLE:** Prevalence of selected circumstances surrounding reported suicide deaths, by age group — National Violent Death Reporting System, Oregon, 2009

	Age group (yrs)							
	
	10–24 (n = 59)		20–44 (n = 193)		45–64 (n = 277)		≥65 (n = 111)	
				
Circumstance	No.	(%)	No.	(%)	No.	(%)	No.	(%)
Depressed mood	16	(27)	67	(35)	93	(34)	33	(30)
Alcohol or substance abuse	14	(24)	66	(34)	80	(29)	10	(9)
Relationship problem	21	(36)	93	(48)	74	(27)	12	(11)
Job or financial problem	6	(10)	52	(27)	82	(30)	7	(6)
Lived alone	NA	NA	NA	NA	NA	NA	49	(44)
Chronic disease or declining health	13	(22)	45	(23)	94	(34)	75	(68)
Went to health-care provider in the 30 days before death	NA	NA	NA	NA	NA	NA	19	(17)
Disclosed suicidal intent	22	(37)	96	(50)	106	(38)	44	(40)

**Abbreviation:** NA = not assessed; these data were collected only for decedents aged ≥65 years.

## References

[1] World Health Organization (2010). Injuries and violence: the facts.

[2] CDC (2013). Web-Based Injury Statistics Query and Reporting System (WISQARS).

[3] CDC (2012). Compendium of effective fall interventions: what works for community-dwelling older adults.

[4] CDC (2012). Trends in suicide rates among persons ages 10 years and older, by sex, United States, 1991–2009.

[5] Joiner T (2005). Why people die by suicide.

[6] Alexander J, Kohn M, Millet L, Moreland S, Pollock D (2006). Oregon older adult suicide prevention plan.

[7] National Highway Traffic Safety Administration (2013). Traffic safety facts, 2011 data: alcohol-impaired driving.

[8] Zaloshnja E, Miller TR, Blincoe LJ Costs of alcohol-involved crashes, United States, 2010.

[9] Moskowitz H, Fiorentino H (2000). A review of the literature on the effects of doses of alcohol on driving related skills.

[10] National Highway Traffic Safety Administration (2001). Legislative history of 08 per se laws.

[11] General Accounting Office (1999). Highway safety: effectiveness of state 0.08 blood alcohol laws.

[12] Shults RA, Elder RW, Sleet DA (2001). Reviews of evidence regarding interventions to reduce alcohol-impaired driving. Am J Prev Med.

[13] Task Force on Community Preventive Services (2001). Recommendations to reduce injuries to motor vehicle occupants: increasing child safety seat use, increasing safety belt use, and reducing alcohol-impaired driving. Am J Prev Med.

[14] National Highway Traffic Safety Administration (2009). Traffic safety facts 2008: a compilation of motor vehicle crash data from the Fatality Analysis Reporting System and the General Estimates System.

[15] Sleet DA, Howat P, Elder R, Maycock B, Baldwin G, Shults R, Verster JC, Pandi-Perumal SR, Ramaekers JG, de Gier JJ (2009). Interventions to reduce impaired driving and traffic injury. Drugs, driving and traffic safety.

[16] World Health Organization (2013). Global status report on road safety 2013: supporting a decade of action.

[17] ChartsBin Legal blood alcohol concentration (BAC) limits around the world.

[18] Degutis LC (2012). The future of injury and violence prevention: where are we going?. J Safety Res.

[19] Rippe JM (2010). Injury prevention: a medical and public health imperative. Am J Lifestyle Med.

